# Influenza Virus Targets Class I MHC-Educated NK Cells for Immunoevasion

**DOI:** 10.1371/journal.ppat.1005446

**Published:** 2016-02-29

**Authors:** Ahmad Bakur Mahmoud, Megan M. Tu, Andrew Wight, Haggag S. Zein, Mir Munir A. Rahim, Seung-Hwan Lee, Harman S. Sekhon, Earl G. Brown, Andrew P. Makrigiannis

**Affiliations:** 1 College of Applied Medical Sciences, Taibah University, Madinah Munawwarah, Kingdom of Saudi Arabia; 2 Department of Biochemistry, Microbiology, and Immunology, University of Ottawa, Ottawa, Ontario, Canada; 3 Cairo University Research Park, Faculty of Agriculture, Cairo University, Giza, Egypt; 4 Department of Pathology and Laboratory Medicine, The Ottawa Hospital, University of Ottawa, Ottawa, Ontario, Canada; University of Iowa, UNITED STATES

## Abstract

The immune response to influenza virus infection comprises both innate and adaptive defenses. NK cells play an early role in the destruction of tumors and virally-infected cells. NK cells express a variety of inhibitory receptors, including those of the Ly49 family, which are functional homologs of human killer-cell immunoglobulin-like receptors (KIR). Like human KIR, Ly49 receptors inhibit NK cell-mediated lysis by binding to major histocompatibility complex class I (MHC-I) molecules that are expressed on normal cells. During NK cell maturation, the interaction of NK cell inhibitory Ly49 receptors with their MHC-I ligands results in two types of NK cells: licensed (“functional”), or unlicensed (“hypofunctional”). Despite being completely dysfunctional with regard to rejecting MHC-I-deficient cells, unlicensed NK cells represent up to half of the mature NK cell pool in rodents and humans, suggesting an alternative role for these cells in host defense. Here, we demonstrate that after influenza infection, MHC-I expression on lung epithelial cells is upregulated, and mice bearing unlicensed NK cells (Ly49-deficient NKC^KD^ and MHC-I-deficient *B2m* ^-/-^ mice) survive the infection better than WT mice. Importantly, transgenic expression of an inhibitory self-MHC-I-specific Ly49 receptor in NKC^KD^ mice restores WT influenza susceptibility, confirming a direct role for Ly49. Conversely, F(ab’)_2_-mediated blockade of self-MHC-I-specific Ly49 inhibitory receptors protects WT mice from influenza virus infection. Mechanistically, perforin-deficient NKC^KD^ mice succumb to influenza infection rapidly, indicating that direct cytotoxicity is necessary for unlicensed NK cell-mediated protection. Our findings demonstrate that Ly49:MHC-I interactions play a critical role in influenza virus pathogenesis. We suggest a similar role may be conserved in human KIR, and their blockade may be protective in humans.

## Author Summary

Influenza virus has developed a number of immune-evasion mechanisms to prolong its survival within the host. Development of functional NK cells is dependent on multiple factors such as the interaction between MHC-I and Ly49 receptors. NK cells that develop in the absence of these interactions are referred to as ‘unlicensed’ and represent up to half of the total number of NK cells. We show that significant MHC-I upregulation on lung epithelial cells following influenza virus infection most likely allows influenza virus to evade detection by licensed NK cells. Importantly, we demonstrate that unlicensed NK cells play a major role in protecting mice from influenza infection. Both Ly49- and MHC-I-deficient mice, which possess unlicensed NK cells, exhibit better survival than WT mice when infected with a lethal dose of influenza virus. Survival of Ly49-deficient mice is associated with reduced viral titers and lung pathology, compared to the infected WT mice. Moreover, disrupting the interaction between MHC-I and inhibitory Ly49 receptors protects WT mice from a lethal influenza virus infection. These results suggest that the so-called unlicensed NK cells, previously characterized as being hyporesponsive, actually possess potent antiviral activity, and are crucial for protection from influenza virus and possibly other viral infections.

## Introduction

Influenza viruses are classified as members of the Orthomyxoviridae family, which are enveloped viruses with a segmented, negative, single-stranded RNA (ssRNA) genome that contains 7–8 gene segments. Structurally, influenza A virus expresses two surface glycoproteins, hemagglutinin and neuraminidase [1, 2]. Influenza A virus can cause severe human illness, including upper and lower respiratory tract infections and pneumonia, and is associated with major human pandemics. Seasonal influenza epidemics result in 250,000–500,000 deaths worldwide annually [3].

NK cells are innate lymphocytes that play a critical role in host defense against tumors and virus infection, both by directly eliminating them and by enhancing the rapid development of adaptive responses [4–6]. NK cells are important for protection against influenza virus infection in various animal models [5, 7, 8]. In response to NK cell cytolytic function, influenza virus has developed several evasion strategies to escape NK cell recognition [9, 10]. Importantly, influenza virus infection was shown to induce accumulation of MHC-I molecules in the lipid raft microdomains of infected cells, leading to increased binding of the NK cell inhibitory receptor KIR2DL1 and inhibition of human NK cell cytotoxicity *in vitro* [11, 12].

NK cell effector functions are tightly controlled by the combination of signals received through germline-encoded activating and inhibitory receptors [6, 13]. Mouse NK receptors include the Ly49, NKG2, and NKR-P1 families of receptors encoded in the Natural Killer gene Complex (NKC) on chromosome 6 [13, 14]. Inhibitory receptors engage molecular indicators of health, while activating receptors engage indicators of disease. By integrating these signals, the NK cell can appropriately spare or destroy a potential target [14]. Ly49 family members are type II transmembrane glycoproteins, part of the C-type lectin superfamily that forms disulphide-linked homodimers [15]. The mouse Ly49 are functionally equivalent to human killer-cell immunoglobulin-like receptors (KIR). The ligands for KIR and Ly49 receptors are self MHC-I molecules or MHC-I related molecules that are expressed by pathogens upon infection [5, 16, 17].

Beyond regulating NK cell killing, interactions between MHC-I and Ly49 receptors are required for NK cell education. The licensing hypothesis states that, to be fully functional, a developing NK cell must successfully engage a self-ligand with an inhibitory receptor [18, 19]. In a C57BL/6 mouse, this is canonically achieved by engagement of MHC-I by Ly49C and/or Ly49I. Accordingly, NK cells that do not express Ly49C/I, or cells from MHC-I-deficient or Ly49-deficient (NKC^KD^) mice, are ‘unlicensed’, displaying attenuated responses to MHC-I-deficient tumors *in vitro* and *in vivo* [19–22]. NKC^KD^ mice also develop lymphomas earlier than WT mice, again suggesting a degree of dysfunction in unlicensed NK cells [22]. Despite being unlicensed, however, these Ly49C/I^-^ cells represent up to half of the population of mature NK cells in a healthy, WT mouse [9, 18, 23–25], suggesting a role for these cells in host defense.

Since these unlicensed cells are dysfunctional with regard to rejecting MHC-I-deficient tumors, their role in host defense may be in NK-mediated anti-pathogen activity. MHC-I-deficient (*B2m* ^-/-^) mice, which bear only unlicensed NK cells, exhibit robust NK cell responses and can control mouse cytomegalovirus (MCMV) infection as efficiently as WT mice [26, 27]. A publication by Orr et al. found that adoptively transferred unlicensed Ly49C/I^-^ Ly49G2^+^ NK cells into MCMV-infected neonates enhanced their survival better than the licensed, Ly49C/I^+^ Ly49G2^+^ cells [28]. For a more in-depth analysis of the role of unlicensed NK cells in viral infection, we have used Ly49-deficient (NKC^KD^) mice generated in our laboratory [20], in which approximately 80% of NK cells are unlicensed. Thus, NKC^KD^ mice serve as a model to study the role of unlicensed NK cells during viral infections. In this study, we explore the interactions of influenza virus with licensed and unlicensed NK cells. We present evidence that influenza effectively evades licensed NK cells, but is eliminated by unlicensed NK cells in a perforin-dependent manner. Importantly, genetic and physical disruption of Ly49 binding to its MHC-I ligands results in enhanced NK cell-mediated control of influenza virus infection *in vivo*, implicating virus-induced MHC-I expression as an immunoevasion strategy.

## Materials and Methods

### Mice

C57BL/6 (B6) and B6.129P2-B2m^tm1^Unc/J (*B2m* ^-/-^) mice were purchased from The Jackson Laboratory (Bar Harbor, ME), *Ifnar1* ^-/-^ were obtained from Dr. Subash Sad (University of Ottawa, Ottawa, ON). B6.NKC^KD^, B6.NKC^KD^-Ly49I^tg^, B6.Ly49Q^KO^, and their congenic control, B6.Ly49^129^ mice, have been previously described [20, 29]. Mice deficient in both Ly49 (NKC^KD^) and perforin (*Prf* ^-/-^) were produced by mating B6.NKC^KD^ with B6.Prf^-/-^ mice. B6.NKC^KD^, B6.Ly49Q^KO^ and WT controls were bred as homozygous pairs. B6.NKC^KD^-Ly49I^tg^ and B6.Prf^-/-^ mice were bred as heterozygous mating pairs, and littermates were used for experimentation. Assessment of genotypes was performed by PCR.

### Ethics statement

All mice were maintained in a specific-pathogen-free environment. All breeding and manipulations performed on animals were approved by the University of Ottawa animal care committee (protocol BMI-2049) in accordance with the principles published in the Canadian Council on Animal Care’s “Guide to the Care and Use of Experimental Animals” and with the Animals for Research Act, R.S.O. 1990, c.22, s. 17(1–3).

### Influenza virus infection

Groups of age and sex-matched mice (6–8 weeks old) were anesthetized with isoflurane and inoculated intranasally with 600 or 1050 PFU of mouse-adapted A/FM/1/47-MA (FM-MA) strain influenza virus [30]. Influenza-infected mice were housed in a level 2 confinement area for the duration of the experiment. Body weight was measured daily. Animals were considered to be at endpoint if weight loss exceeded 25% of the body weight prior to infection, or if the animal was moribund. Viral loads of infected mice were determined by plaque assay, as described previously [30]. Virus titer is expressed as the number of plaque forming units per gram of lung (PFU/g).

### *In vivo* mAb treatments

Anti-NK1.1 mAb (clone PK136), anti-IFN-γ mAb (clone XMG1.2), and anti-Ly49C/I F(ab')_2_ mAb (clone 5E6) were injected i.p. into groups of age and sex-matched WT mice. 200 μg of mAb per mouse were injected i.p. two days prior to influenza virus infection, on the day of infection, and every two days post-infection until day 10 p.i. Anti-AsialoGM1 antibody (Wako Pure Chemical Industries, Osaka, Japan) was injected i.p. two days prior to influenza virus infection (25 μl), on the day of infection (25 μl), and every three days post-infection (10 μl) until day 10 p.i.

### Lung epithelial cell isolation and staining

Lungs were removed and minced in 5 ml RPMI with 0.5 mg/ml collagenase D (Roche), followed by incubation for 1 h at 37°C with agitation. The minced pieces were crushed on a 70 μm cell strainer to prepare single cell suspensions for flow cytometry as previously described.

### Antibodies and flow cytometry

Anti-mouse CD18 (LFA-1), CD326 (EpCAM), MHC-I (H-2K^b^), 5E6 (anti-Ly49C/I), 4D11 (anti-Ly49G), CD8 (CD8β), CD4, CD3, TCRβ, NKp46 (CD335), NKG2D (CD314), NKG2A (16a11), NKG2A/C/E (20d5), CD27, CD11b, CD107a (1D4B), IFN-γ (XMG1.2), and Live/Dead stain were purchased from eBioscience (eBioscience, San Diego, CA, USA). Anti-NKG2D (CD314) was purchased from BioLegend (BioLegend, San Diego, CA, USA). Anti-mouse TCRβ chain was purchased from BD Biosciences (BD Biosciences, Mississauga, Ontario, Canada). PK136 (anti-NK1.1), 5E6 (anti-Ly49C/I^B6^), and XMG1.2 (anti-IFN-γ) hybridomas were kind gifts from Drs. James Carlyle (Sunnybrook Research Institute, Toronto, ON), Charles Sentman (Dartmouth Hitchcock Medical Center, Lebanon, New Hampshire), and Subash Sad (University of Ottawa, Ottawa, ON), respectively. Cell fluorescence data was acquired with a CyAN-ADP flow cytometer (Beckman Coulter) and analyzed with Kaluza software (Beckman Coulter, New Jersey, USA).

The levels of cytokines and chemokines in lung tissue homogenates were measured by bead array flow cytometry using the mouse Th1/Th2/Th17/Th22-13plex FlowCytomix multiplex kit and mouse chemokine 6plex kit (eBioscience, San Diego, CA, USA).

### Tetramer staining

Streptavidin-PE conjugated influenza A non-structural protein (NS2)_114-121_ (RTFSFQLI) and nucleocapsid protein (NP)_311-325_ (QVYSLIRPNENPAHK) tetramers were kindly provided by the NIH Tetramer Core Facility at Emory University (Emory University Vaccine Center, Atlanta, GA). 5x10^5^ lung cells were stained with 1 μg of tetramer in 20 μL of cRPMI and incubated for 1 h at 37°C. Influenza virus-specific CD8^+^ T cells were stained with H-2K^b^NS2_114-121_ (RTFSFQLI) tetramer, while influenza virus-specific CD4^+^ T cells were stained with the I-A^b^ (NP)_311-325_ (QVYSLIRPNENPAHK) tetramer.

### *In vitro* NK cell assay

Total lymphocytes isolated from the lungs were incubated with YAC-1 cells at 1:1 ratio or with phorbol 12-myristate 13-acetate (PMA, 10 μg/ml) and ionomycin (1 μg/ml) in the presence of anti-CD107a mAb and brefeldin A (eBiosience) for 4 h. Cells were stained for surface markers followed by intracellular staining for IFN-γ using IC fixation and permeabilization reagents (eBioscience) following manufacturer’s instructions.

### Purification and modification of mAbs

Individual hybridoma clones were cultured in DMEM supplemented with 1 mM sodium pyruvate, 0.1 mM non-essential amino acids, 0.1 mM β-mercaptoethanol, 100 U/ml penicillin, and 100 μg/ml streptomycin. Culture supernatants were then centrifuged (10,000x g for 20 min) and filtered through a 0.45 μm filter. Monoclonal antibodies (mAb) were purified using Protein G sepharose chromatography (ExalphaBiologicals, Inc, USA). Monoclonal Ab was dialyzed against 1x PBS buffer (pH 7.4) and then concentrated using an Amicon ultra-15 centrifugal filter unit with an ultracel-100 kDa membrane (EMD Millipore Corporation, MA, USA). Monoclonal Ab concentration was determined by SDS-PAGE gel and by spectrophotometric measurement at 280 nm.

To make 5E6 F(ab')_2_ fragments [31], mAb was dialyzed twice against 100 mM sodium acetate solution (pH 4.0), digested using pepsin (Sigma-Aldrich, Ontario, Canada), and then dissolved in 100 mM sodium acetate solution (pH 4.0) at a 1:40 pepsin to mAb ratio for 10 h at 37°C. The digested mAb was dialyzed against 1x PBS buffer (pH 7.4) and then concentrated using an Amicon ultra-15 centrifugal filter unit with an ultracel-50 kDa membrane (EMD Millipore Corporation, MA, USA). F(ab')_2_ fragments were then purified using protein A affinity chromatography. 5E6 F(ab')_2_ fragment concentration was determined by SDS-PAGE gel and by spectrophotometric measurement at 280 nm. The purity of 5E6 F(ab')_2_ fragments was determined by SDS-PAGE gel.

### Lung histopathology

Lungs were collected from infected mice 7 days p.i. and fixed in 10% neutral buffered formalin (25 ml) for 24 h. Subsequently, lungs were embedded in paraffin, sectioned at a thickness of 4 μm and stained with hematoxylin and eosin (H&E). Slides were examined under a microscope to score histopathologic changes in the lungs by a pathologist blind to the experimental conditions.

### Statistical analysis

Statistical comparisons were made by a two-tailed Student’s t-test, one-way ANOVA with Bonferroni post-hoc test, or Kaplan Meier survival statistical analysis (log rank test) using GraphPad Prism software (GraphPad, San Diego, USA). A *p* value <0.05 was considered statistically significant.

## Results

### Ly49-deficient mice are protected from lethal influenza virus infection

**Fig 1:**
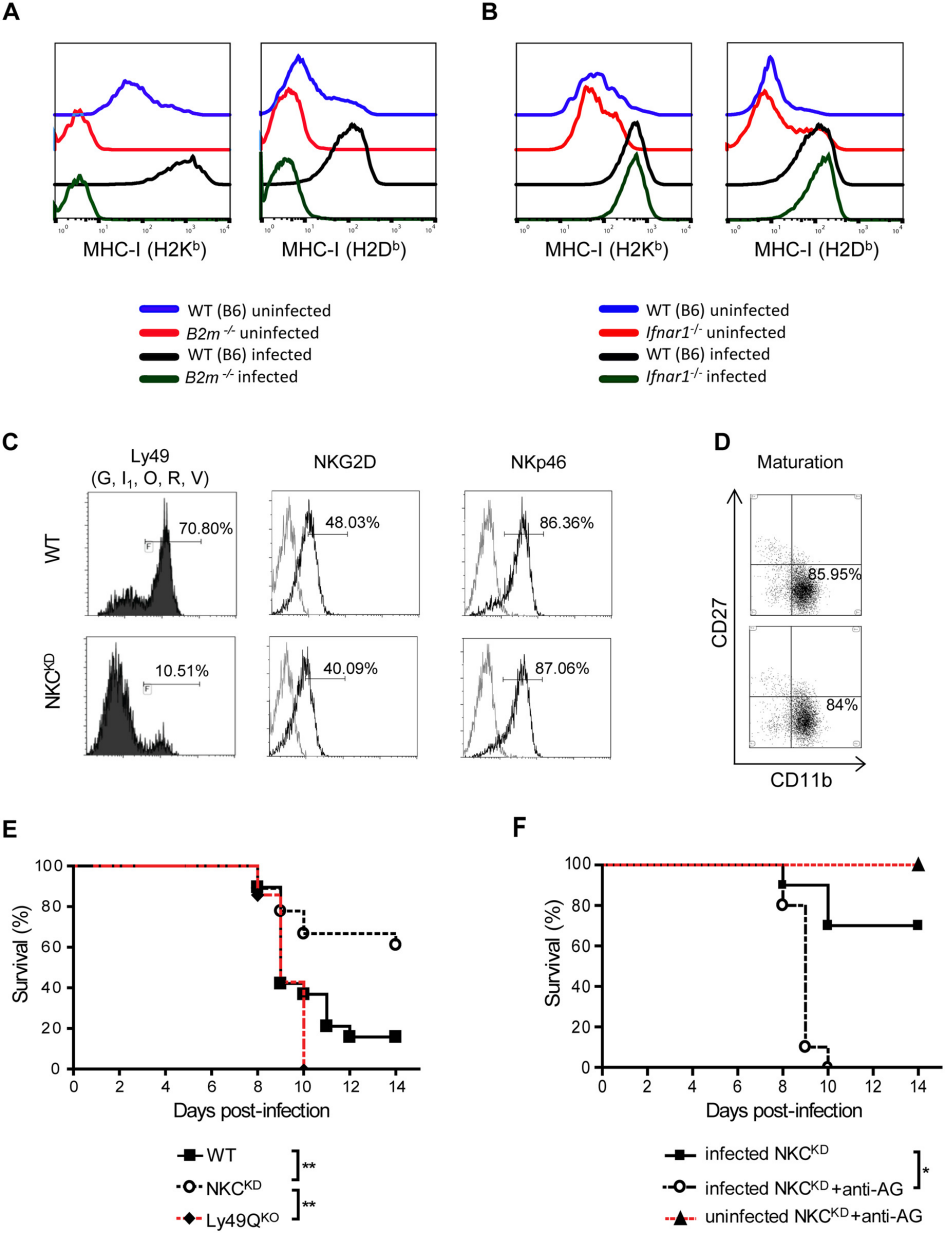
Ly49-deficient mice are protected from lethal influenza infection. **(A, B)** Groups of sex-matched WT (B6), *Ifnar1* ^-/-^ and *B2m* ^-/-^ mice were infected intranasally with 600 PFU of FM-MA virus. Single-cell suspensions were prepared from uninfected lungs and those infected with the virus for 5 days. Cells were stained with antibodies against H-2K^b^ or H-2D^b^ and CD326 (EpCAM—epithelial cell marker), and analyzed by flow cytometry. Surface expression of H-2K^b^ or H-2D^b^ was determined on EpCAM^+^ lung epithelial cells. The following mAb were used in this experiment: anti-LFA-1, anti-EpCAM, anti-H-2K^b^, and anti-H-2D^b^. One representative image from each group is shown. This experiment was performed three times with similar results. **(C, D)** Ly49G, I_1,_ O, R, and V expression was detected on lung NK cells of uninfected WT and NKC^KD^ mice using anti-NKp46, anti-TCRβ, and a combination of 4D11, 4E5 and 14B11 mAb. NKG2D, NKp46, CD11b, and CD27 expression was detected on lung NK cells, defined using anti-CD49b (DX5) and anti-TCRβ. The gray line represents staining with an isotype antibody. This experiment was performed three times with similar results. **(E)** Groups of age and sex-matched WT, Ly49Q^KO^, and NKC^KD^ mice were infected with FM-MA virus (1050 PFU) and monitored for 2 weeks. Data are pooled from two independent experiments (n = 19 in each group). **(F)** Groups of age and sex-matched NKC^KD^ mice with or without NK depletion by anti-asialoGM1 were infected and monitored as above. A group of uninfected, NK-depleted mice was included as a control (n = 10 in each group). The percentage of surviving mice is shown. *p < 0.05, **p < 0.01 and ***p < 0.001. Statistical analysis was performed with the log rank test.

It is well established that influenza virus infection *in vitro* inhibits NK cell cytotoxicity by enhancing NK cell inhibitory receptor binding to MHC-I on infected human lung epithelial cells [11, 12]. It is possible that MHC-I has direct negative effects on NK cell activity during influenza virus infection in mice as well. To test this, we first determined whether influenza virus infection could modulate MHC-I expression on mouse lung epithelial cells. WT (B6) mice were infected with 600 PFU of mouse-adapted influenza strain A/FM/1/47 (H1N1) (FM-MA) intranasally. MHC-I expression was determined on EpCAM^+^ (CD326) lung epithelial cells on day 5 post-infection (p.i.) by flow cytometry. In uninfected mice, low levels of MHC-I expression were detected on lung epithelial cells (Fig 1A). However, upon infection with influenza virus, lung epithelial cell expression of MHC-I was dramatically increased (Fig 1A). As a control, infected and uninfected MHC-I-deficient mice (*B2m* ^-/-^ mice) were included, and as expected, displayed no increase in MHC-I staining upon infection. Influenza virus infection is known to upregulate type I IFN (IFN-I) production, which in turn drives the upregulation of a variety of immunomodulatory proteins including MHC-I. To determine whether this upregulation is mediated by IFN-I production, we infected WT (B6) and mice deficient in the receptor for IFN-α and IFN-β (*Ifnar1* ^-/-^) with 600 PFU FM-MA virus intranasally. Lungs were harvested on day 5 p.i. Interestingly, expression levels of MHC-I were similar between WT (B6) and *Ifnar1* ^-/-^ mice (Fig 1B), confirming this upregulation was independent of IFN-I. These data demonstrate that influenza virus infection induces upregulation of MHC-I expression on lung epithelial cells. Increased expression of MHC-I on lung epithelial cells upon influenza virus infection may have implications for the inhibition of NK cells through interaction with inhibitory Ly49 receptors.

Recently, we reported that Ly49-deficient (NKC^KD^) mice exhibit uncontrolled tumor growth and metastases [22]. Lacking licensed NK cells renders these mice highly susceptible to tumor formation, despite having otherwise normal mature NK cells. Flow cytometry analysis of lung lymphocytes showed that NK cells in the lungs of NKC^KD^ mice were mostly devoid of Ly49 expression (Fig 1C), but were otherwise predominantly mature cells (CD11b^+^ CD27^low^) with normal expression of the activating receptors NKp46 and NKG2D (Fig 1C and 1D). To determine whether Ly49 interaction with MHC-I molecules is relevant to influenza infection *in vivo*, we inoculated WT and NKC^KD^ mice with 1050 PFU of FM-MA intranasally. The animals were observed daily for over two weeks, and sacrificed when moribund. Death due to infection began occurring on day 8 p.i. (Fig 1E). Two weeks p.i., almost 90% of the WT mice had succumbed to the infection, while unexpectedly, only 35% of the NKC^KD^ mice succumbed (Fig 1E, **p = 0.0072). Next, we depleted NK cells in our mouse model to confirm the role of NK cells during influenza virus infection. This depletion was performed with anti-AsialoGM1 treatment instead of the standard NK1.1 treatment, as these mice express an allele of NKR-P1C that is not recognized by the NK1.1 antibody. Anti-AsialoGM1 is known to deplete some activated macrophages and CD8^+^ T cells at high doses [32, 33]. We used a dose that showed total NK depletion without T cell depletion (S1A Fig); however, depletion of activated T cells and activated macrophages remains a possibility. Depletion of NK cells from NKC^KD^ mice using anti-AsialoGM1 treatment (S1B Fig) resulted in a complete loss of protection (Fig 1F), indicating that NK cells from NKC^KD^ mice play a key role in protecting those mice from influenza. Very low (50 PFU) or high (5000 PFU) doses were too extreme to note any survival differences, but 600 PFU gave similar results to 1050 PFU (S2A Fig, Fig 1E and 1F), therefore, 600 PFU infection dose was used for the remainder of the study.

NKC^KD^ mice lack Ly49Q, which plays an important role in IFN-α production by plasmacytoid dendritic cells [29, 34]. To confirm that this survival advantage is not due to a loss of Ly49Q alone, we inoculated Ly49Q^KO^ mice with influenza virus. Like WT mice, Ly49Q^KO^ mice died 10 days post influenza virus infection (Fig 1E, **p = 0.0049). NKC^KD^ mice also express lower levels of NKG2A/C/E. However, the inhibitory NKG2A is not believed to be involved in influenza protection by NK cells [35], and its expression is not altered in *B2m* ^-/-^ mice (S3A Fig), which are also protected from influenza virus (described below). In addition, while NKG2A/C/E expression is decreased in NKC^KD^ mice, it is increased in *B2m* ^-/-^ mice (S3B and S3C Fig). These data show that NKC^KD^ mice survive influenza virus infection better than WT mice in a Ly49Q- and NKG2A/C/E-independent manner. Better survival of NKC^KD^ mice compared to WT mice indicates a possible role for Ly49:MHC-I interactions in the pathogenesis of influenza virus in mice.

### Higher numbers of unlicensed NK cells may confer survival advantage in Ly49-deficient mice

**Fig 2:**
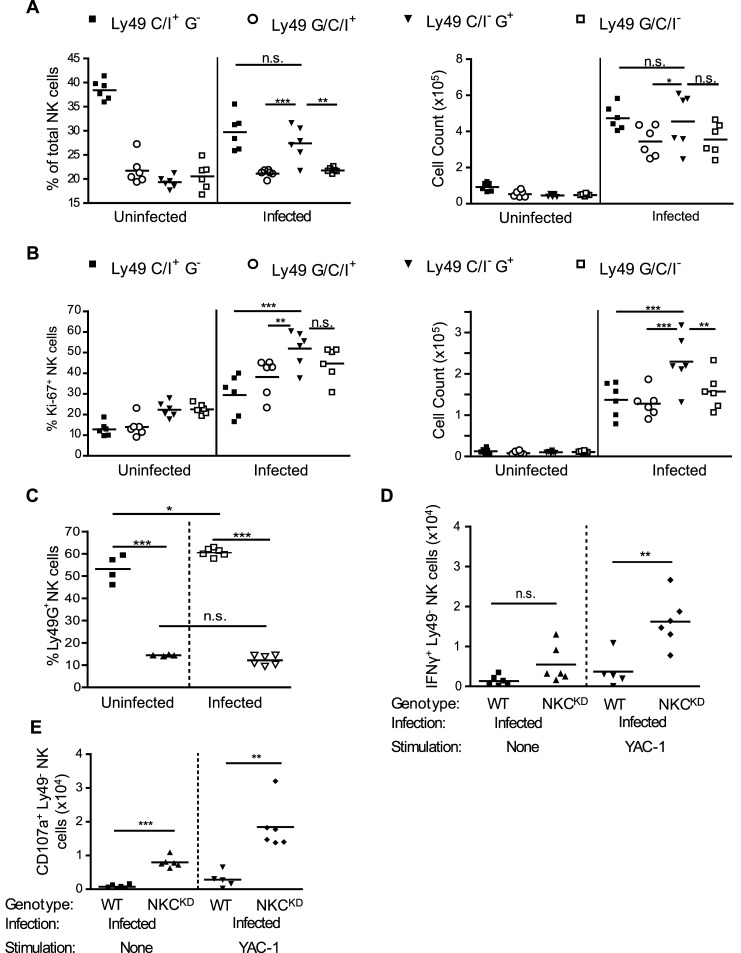
Increased numbers of activated, uneducated NK cells correlates with protection from influenza. Groups of age and sex-matched WT mice were infected as in Fig 1. On day 5 p.i., mice were sacrificed, and single cell suspensions prepared from the lungs. Flow cytometry was performed as above, using cells stained with a fixable viability dye, anti-NKp46, anti-TCRβ, anti-Ly49C/I (5E6), anti-Ly49G (4D11), anti-Ki-67, anti-IFN-γ, and anti-CD107a mAbs. Each symbol represents one mouse. Cell counts are given in absolute number calculated per lung. Horizontal bars represent mean values, *p < 0.05, **p < 0.01, and ***p < 0.001. Statistical analysis was performed using a one-way ANOVA followed by a Bonferroni post-hoc test.

Our data implicate NK cells from NKC^KD^ mice, which are unlicensed due to a lack of Ly49 receptors, in better protection against influenza. To determine the responsiveness of unlicensed NK cells during influenza virus infection, we compared the function of different NK cell subsets in WT mice following infection. NK cells in WT mice can be divided into four populations based on their expression of Ly49C/I and Ly49G. While the licensed Ly49C/I^+^ G^-^ population dominates the lung microenvironment in the steady-state (Fig 2A, left), following infection, the unlicensed Ly49C/I^-^ G^+^ population shows more dramatic expansion (Fig 2A, right). This can be attributed in part to the greater number of proliferating cells in this subpopulation following infection (Fig 2B). NK cells were activated (IFN-γ^+^ and CD107a^+^) upon infection with influenza virus, however, we observed equal levels of intracellular IFN-γ levels in all of these four NK cell subsets (S4 and S5 Figs). While we see an outgrowth of the Ly49G^+^ cells in the WT mice following infection, we observe no similar outgrowth from the residual Ly49G^+^ cells present in NKC^KD^ mice (Fig 2C), indicating that it is likely a lack of Ly49C/I, and not the presence of Ly49G, that is conferring the survival advantage in these animals.

Ly49-negative NK cells are not subject to inhibition via interaction with MHC-I molecules. A comparison of intracellular IFN-γ levels and CD107a (LAMP1) expression on this subset of NK cells in the WT and NKC^KD^ mice revealed a similar level of response in both mice following influenza infection (S6A and S6B Fig). However, a significantly higher number of IFN-γ^+^ and CD107a^+^ NK cells lacking expression of Ly49 are present in the NKC^KD^ mice, due to the disruption of Ly49 expression in these mice (Fig 2D and 2E). Therefore, the presence of a larger number of activated NK cells, which are not inhibited via interaction with the increased expression of MHC-I molecules on the lung epithelial cells in the influenza-infected mice (Fig 1A), may confer a survival advantage to NKC^KD^ compared to the WT mice, during an influenza virus infection.

### WT mice exhibit higher lung viral loads in comparison to NKC^KD^, resulting in severe lung immunopathology, following lethal influenza infection

**Fig 3:**
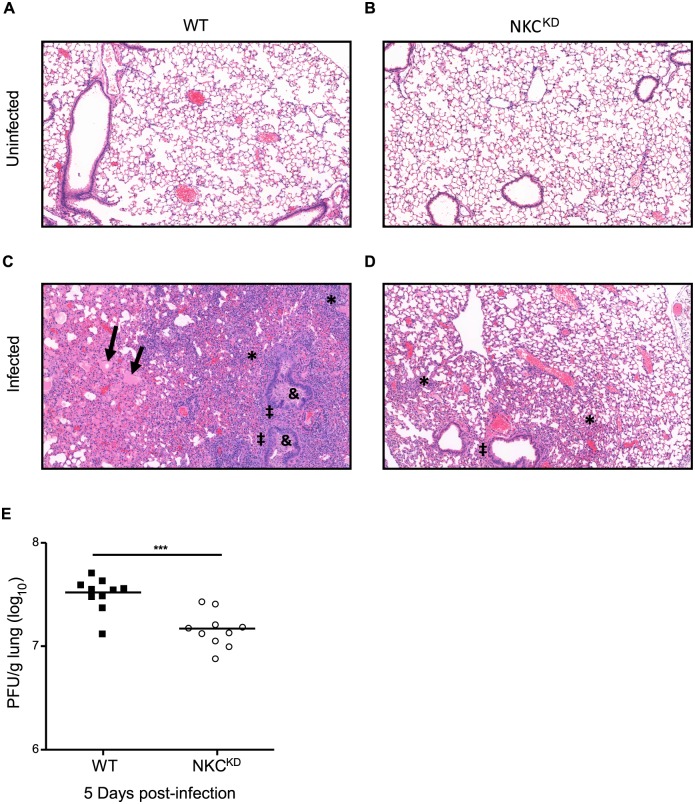
Lung immunopathology of influenza-infected WT and NKC^KD^ mice. **(A-D)** WT and NKC^KD^ mice were inoculated intranasally with 600 PFU of FM-MA virus. Lungs were collected from WT and NKC^KD^ mice 7 days p.i., fixed in neutral-buffered 10% formalin, sectioned, and stained with H&E. Images were acquired at 100x magnification. One representative image from each group is shown. Images were scored by a pathologist blind to the experimental conditions. Regions of tissue damage are indicated as follows: ‘→’ pulmonary edema; ‘*’ diffuse alveolar damage; ‘‡’ lymphocytic and neutrophilic infiltrate; ‘&’ bronchi filled with cellular debris. This experiment was performed three times with similar results. Two to three mice were used for each group per experiment. **(E)** Lungs from infected (600 PFU) age- and sex-matched WT and NKC^KD^ mice were collected on day 5 p.i., weighed, and virus titer (presented as PFU/g of lung tissue) was assessed in lung homogenates by plaque assay on MDCK cells. Data are pooled from three independent experiments (n = 10 in each group). Each symbol represents a single mouse. Horizontal bars represent mean values. ***p < 0.001. Statistical analysis was performed using Student’s t-test.

Influenza virus infection causes severe lung pathology, leading to respiratory distress and mortality [36]. To examine lung pathology in WT and NKC^KD^ mice, lungs were collected 7 days p.i. with 600 PFU of FM-MA viruses. Microscopic examination of H&E stained lung sections showed more severe alveolar damage, leukocyte infiltration, and pulmonary edema in WT mice compared to NKC^KD^ mice (Fig 3A–3D). Similar results were obtained with 1050 PFU (S2B Fig). From these data, we attribute the increased mortality in WT mice over NKC^KD^ to more severe influenza-induced—and possibly immune-mediated—lung pathology. Next, we asked whether NKC^KD^ mice can eliminate influenza virus-infected cells more efficiently than WT mice, possibly leading to less viral burden and inflammation. To avoid survivor bias and ensure that the observed effect is due to innate immune responses, lungs were collected from infected WT and NKC^KD^ mice 5 days p.i., and viral titers were determined. Interestingly, viral titer in NKC^KD^ mice was significantly lower than in WT mice on day 5 p.i. (Fig 3E), while viral loads were equivalent between the two on days 3 and 8 p.i. (S7A Fig). This result indicates that NKC^KD^ mice are better than WT mice at controlling lung viral loads early during infection. Better control of the virus may lead to lower levels of inflammation and decreased lung injury following influenza virus infection in NKC^KD^ mice.

### Ly49 deficiency results in significantly decreased levels of pro-inflammatory cytokine and chemokine levels in the lungs of NKC^KD^ mice after influenza virus infection

**Fig 4:**
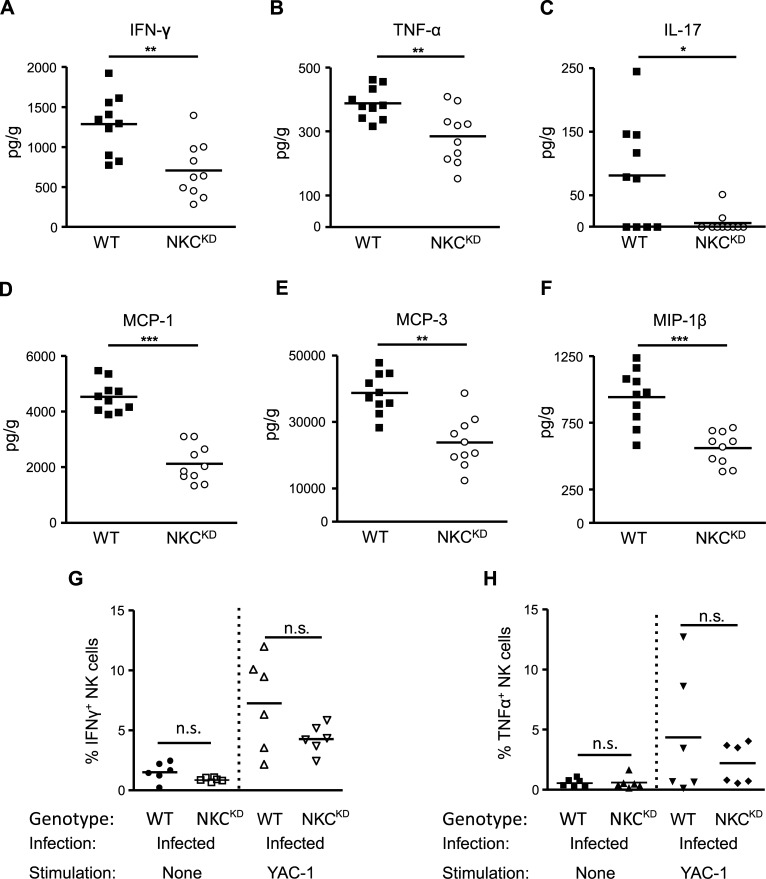
Cytokine and chemokine levels in the lungs of influenza virus-infected mice. Groups of age and sex-matched WT and NKC^KD^ mice were infected intranasally with 600 PFU of FM-MA virus. Lungs were collected on day 5 p.i., and lung tissue homogenates were prepared. The levels of **(A)** IFN-γ, **(B)** TNF-α, **(C)** IL-17, **(D)** MCP-1, **(E)** MCP-3, and **(F)** MIP-1β in the lung tissue homogenates were measured by flow cytometry using FlowCytomix Multiplex kits. Data are pooled from three independent experiments. **(G, H)** NK cells were isolated from infected lungs, stained, and analyzed by flow cytometry as above. Cells were stained with a fixable viability dye, anti-NKp46, anti-TCRβ, and anti-IFN-γ **(G)** or anti-TNF-α **(H)**. Horizontal bars represent mean values. Each symbol represents data from a single mouse. *p < 0.05, **p < 0.01 and ***p < 0.001. Statistical analysis was performed using Student’s t-test.

In addition to injury resulting from influenza virus replication, pro-inflammatory cytokines and chemokines have been suggested to play a pathogenic role in humans and animals infected with influenza virus [37, 38]. We have demonstrated previously that, similar to WT mice, NK cells from NKC^KD^ mice produce normal levels of cytokines upon stimulation with tumor cell lines, anti-NKp46 mAb, and after murine CMV (MCMV) infection [20]. To address the role of pro-inflammatory cytokines and chemokines in the pathogenesis of influenza viruses, we determined the cytokine and chemokine profile in the lungs of influenza-infected WT and NKC^KD^ mice by bead array flow cytometry, 5 days p.i. The majority of cytokines had similar baseline levels in WT and NKC^KD^ mice (S8 Fig). However, the most striking differences occurred in the levels of TNF-α, IFN-γ, IL-17, MCP-1, MCP-3, and MIP-1β, which were elevated significantly in lung homogenates of WT mice compared to the NKC^KD^ mice (Fig 4A–4F). These remarkable changes in pro-inflammatory cytokines and chemokines in the lungs of influenza virus-infected WT mice suggest their involvement in lung pathology. Furthermore, while we noticed a trend toward elevated IFN-γ and TNF-α in the WT lung NK cells themselves (Fig 4G and 4H), it is likely that other immune cells in the microenvironment are contributing to the cytokine profile we observe in the bulk lung extracts.

### Early NK but not influenza virus-specific T cell increase in the lungs of influenza virus-infected mice

**Fig 5:**
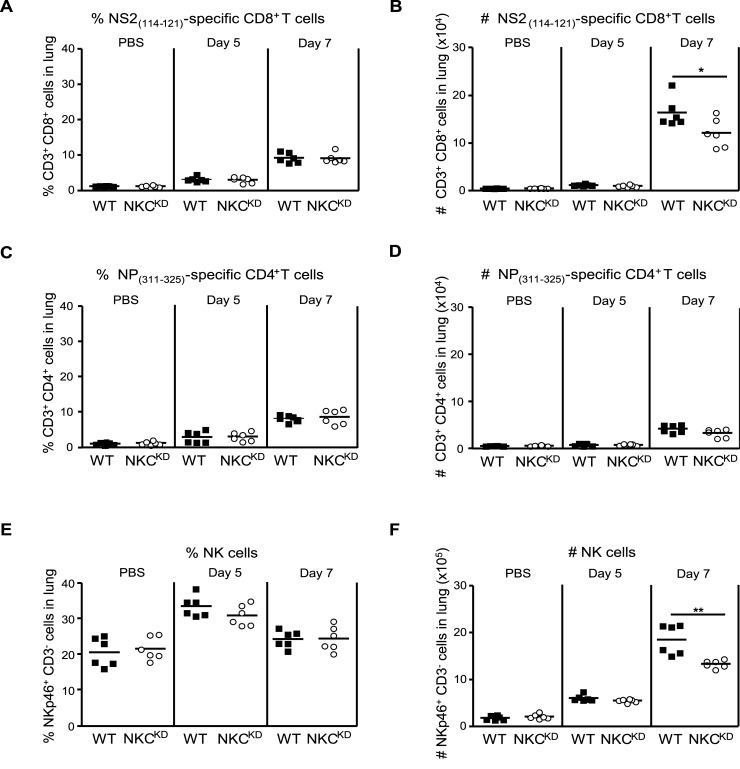
Lymphocyte recruitment to the lungs following influenza virus infection. WT and NKC^KD^ mice were infected intranasally with 600 PFU of FM-MA virus. Single-cell suspensions were prepared from uninfected lungs and those infected with the virus for 5 or 7 days. The following mAb were used in this experiment: anti-CD8, anti-CD3, anti-CD4, and anti-NKp46 mAb; as well, K^b^-NS2_114−121_ tetramers and I-A^b^–NP_311-325_ tetramers were used. Both the frequency (%) and absolute number (#) of cell populations is reported. **(A, B)** NS2_114-121_ CD8^+^ specific T cells were gated out of CD3^+^ CD8^+^ T cells. **(C, D)** NP_311-325_ CD4^+^specific T cells were gated out of CD3^+^ CD4^+^ T cells. **(E, F)** NK cells were detected as CD3^-^ NKp46^+^ cells. Data are pooled from two independent experiments (n = 6 in each group). Horizontal bars represent mean values. Each symbol represents data from a single mouse. *p < 0.05 and **p < 0.01. Statistical analysis was performed using Student’s t-test.

To demonstrate that the observed significant differences in cytokine levels and influenza virus load in the lungs of WT mice at day 5 p.i. is dependent on an NK cell response, we quantified the immune cell subsets responding to viral infection. We examined the NK cell and the virus-specific CD8^+^ and CD4^+^ T cell responses in the lungs of influenza virus−infected WT and NKC^KD^ mice 5 and 7 days p.i. Assessment of the percentage and absolute number of these lymphocyte subpopulations after influenza virus infection showed that a protective response to the infection within the first 5 days directly correlated with NK cell expansion (Fig 5A–5F), and not that of virus-specific CD4^+^ or CD8^+^ T cells. Substantial CD4^+^ and CD8^+^ T lymphocyte counts were only observed 7 days post-influenza virus infection (Fig 5A–5D); however, flow cytometry detected an expansion of the NK cell population in all mice 5 days p.i. (Fig 5E–5F). Notably, the percentage and the number of NK cells from both WT and NKC^KD^ mice show that there is no statistically significant difference between both groups 5 days p.i. However, the number of NK cells was significantly higher in WT mice (**p = 0.0031) compared to NKC^KD^ mice (Fig 5F) 7 days p.i., most likely as a result of the reduction in virus load along with cytokine levels in the lungs of NKC^KD^ mice at day 5. These data strongly suggest that activation and increase in NK cell numbers within the first 5 days post-influenza virus infection enhances the antiviral response mediated by NK cells, and as a result, plays a substantial role in the initial control of influenza virus, especially by NKC^KD^ mice.

### Direct contribution of Ly49 inhibitory receptors in the pathogenesis of influenza virus

**Fig 6:**
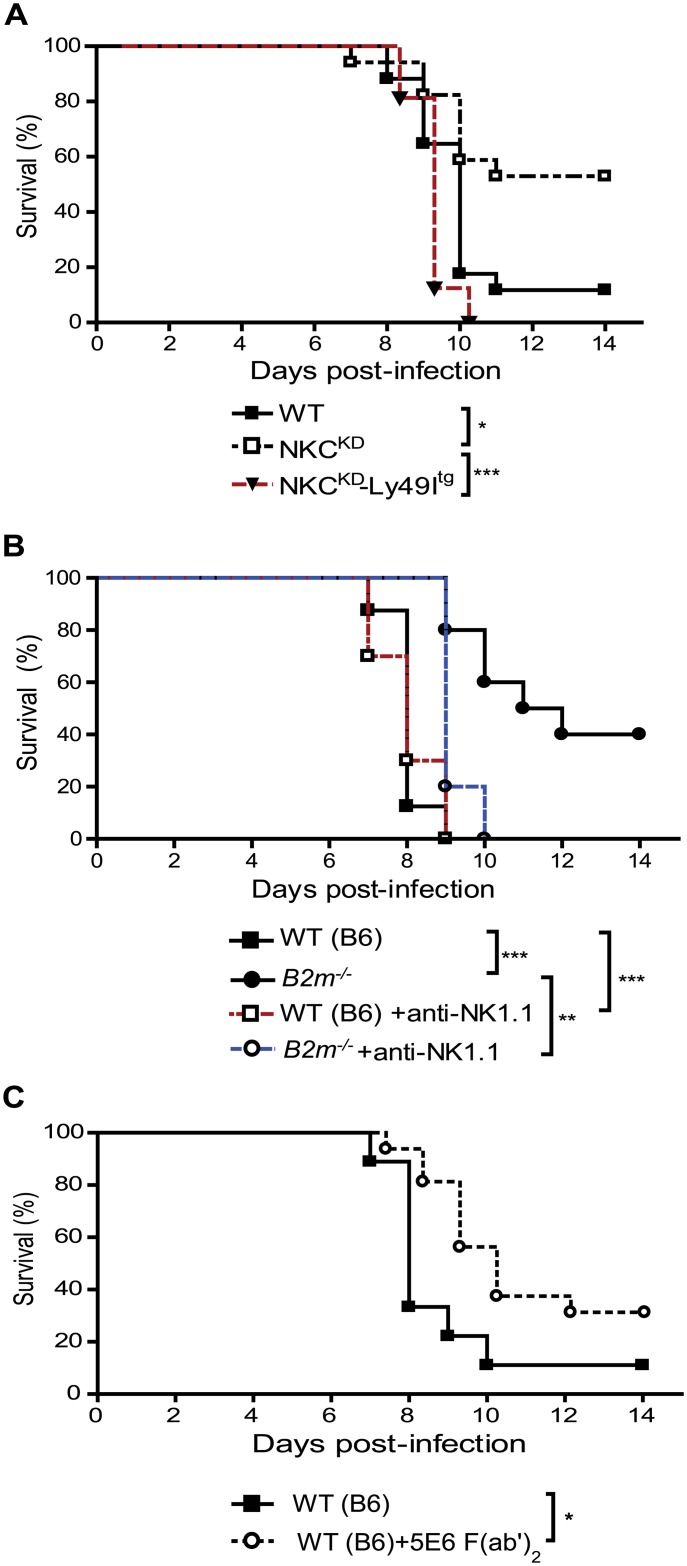
Involvement of Ly49 inhibitory receptors in the pathogenesis of influenza virus. **(A)** Groups of influenza-infected age and sex-matched WT, NKC^KD^, and littermate NKC^KD^-Ly49I^tg^ mice were infected with 600 PFU of FM-MA virus, then monitored as before. Data are pooled from two independent experiments (n = 16 in each group). **(B)** Groups of influenza-infected age and sex-matched WT (B6) and *B2m* ^-/-^ mice were monitored as in Fig 1. One group each of WT or *B2m* ^-/-^ mice was treated with 200 μg anti-NK1.1 mAb per mouse i.p. two days prior to influenza virus infection, on the day of infection (600 PFU), and every two days after until day 10 p.i. Data are pooled from two independent experiments (n = 10 in each group). **(C)** Groups of age and sex-matched WT (B6) mice were treated or not with 200 μg of 5E6 F(ab')_2_ mAbs per mouse i.p. two days prior to influenza virus infection (600 PFU), on the day of infection, and every two days post-influenza virus infection until day 10 p.i. Data are pooled from two independent experiments (n = 16 in each group). *p < 0.05, **p < 0.01 and ***p < 0.001. Statistical analysis was performed with the log rank test.

To test the direct contribution of self-MHC-I-specific Ly49 inhibitory receptors in the pathogenesis of influenza virus, we introduced the inhibitory self-MHC-I-specific Ly49I transgene into NKC^KD^ mice through breeding as previously described [20]. We inoculated WT, NKC^KD^, and NKC^KD^-Ly49I^tg^ mice with 600 PFU FM-MA virus intranasally. The animals were observed daily for over two weeks and sacrificed when moribund. Death due to infection began occurring on day 8 p.i. (Fig 6A). In agreement with our previous results, 90% of WT and 40% of NKC^KD^ mice succumbed by two weeks p.i. (Fig 6A, *p = 0.0147). Remarkably, 100% of NKC^KD^-Ly49I^tg^ mice died ten days p.i. (Fig 6A, ***p<0.0001). Thus, Ly49 deficiency was definitively protective in NKC^KD^ mice, likely via the loss of MHC-I-specific NK cell inhibition. This finding raises an intriguing question as to whether MHC-I-deficient mice would also be protected from lethal influenza virus infection. To answer this question, we infected four groups of mice: WT (B6) and *B2m* ^-/-^ (MHC-I deficient), and WT and *B2m* ^-/-^ mice treated with anti-NK1.1 mAb to deplete NK cells. Remarkably, 50% of *B2m* ^-/-^ mice survived the infection, whereas all B6 mice and NK cell-depleted B6 mice died ten days p.i. (Fig 6B, ***p<0.0001). *B2m* ^-/-^ survival advantage was observed despite a similar viral load measured in the lungs of these mice on day 5 and 7 p.i. compared to the B6 mice (S7B Fig). NK cell activity (IFN-γ^+^ and CD107a^+^) following *in vitro* stimulation was also similar in influenza virus-infected *B2m* ^-/-^ and WT (B6) mice (S6C and S6D Fig); however, NK cell inhibition via Ly49:MHC-I interaction is disrupted in *B2m* ^-/-^ mice. Interestingly, all NK cell-depleted *B2m* ^-/-^ mice succumbed to the infection as well (Fig 6B, ***p = 0.0015), indicating a direct role for MHC-I-unlicensed NK cells in controlling influenza virus infection in these mice. To validate this proof-of-concept, several groups have shown that blocking the interaction between Ly49C/I and their ligands enhances NK-mediated anti-cancer cytotoxic functions [39]. Likewise, it is possible that a functional blockade of Ly49C/I:MHC-I interactions might protect WT (B6) mice during influenza virus infection. To determine whether Ly49 interactions with MHC-I molecules are relevant to influenza infection *in vivo*, WT (B6) mice were treated two days prior to FM-MA infection, at the day of infection, and every two days after until day 10 p.i. with 200 μg of 5E6 F(ab')_2_ mAbs, previously reported to block Ly49C/I:H-2K^b^ interactions [39]. Blockade of Ly49C/I inhibitory receptors resulted in a significant increase in mouse survival when compared with untreated B6 mice (Fig 6C, *p = 0.0313). These results demonstrate that preventing Ly49 inhibitory receptor interactions with their cognate MHC-I ligands is protective in mice against influenza virus infection.

### NK cell-mediated early defense against influenza virus infection requires both IFN-γ and perforin

Our data strongly suggest that an early increase in NK cell numbers in the lungs during influenza virus infection enhances the antiviral response and the initial control of influenza virus, especially in NKC^KD^ mice. NK cells can directly limit virus replication by killing infected cells via the release of cytotoxic granules containing perforin and granzymes, or indirectly by producing IFN-γ, which plays a role in macrophage activation and in inhibiting viral replication.

**Fig 7:**
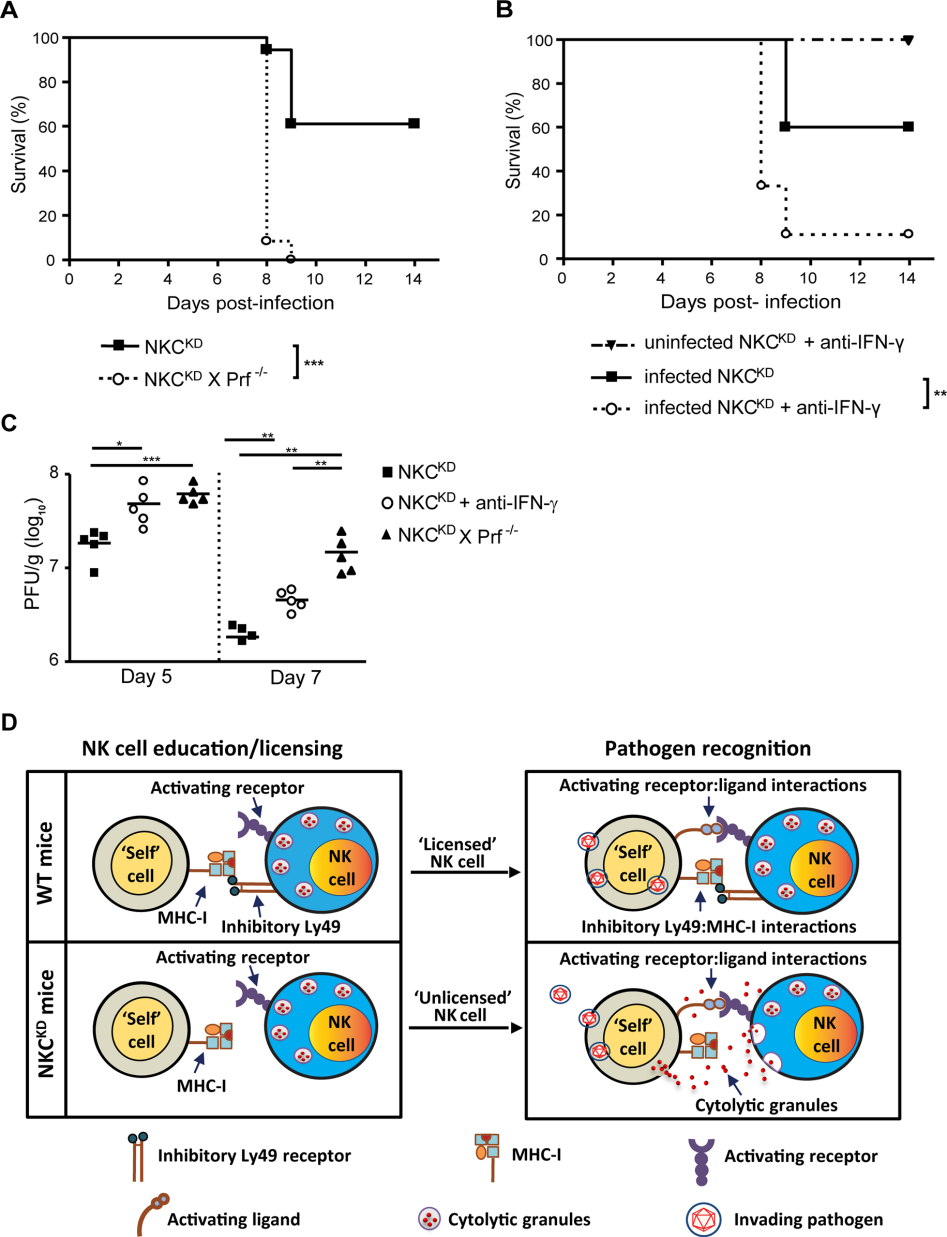
IFN-γ and perforin are required for protection against influenza virus infection. **(A)** Survival of infected NKC^KD^ (n = 18) and NKC^KD^-Prf^-/-^ (n = 12) mice (600 PFU) was monitored as before. Data are pooled from two independent experiments. **(B)** Groups of influenza virus-infected and uninfected NKC^KD^ mice (600 PFU) were treated or not with 200 μg anti-IFN-γ mAb per mouse i.p. two days prior to influenza virus infection, on the day of infection, and every two days post-influenza virus infection until day 10 p.i. Data are pooled from two independent experiments, with the following groups: infected NKC^KD^+anti-IFN-γ (n = 18), infected NKC^KD^ (n = 10), and uninfected NKC^KD^+anti-IFN-γ (n = 6). *p < 0.05, **p < 0.01 and ***p < 0.001. Statistical analysis was performed with the log rank test. **(C)** Lungs from infected (600 PFU) age- and sex-matched WT and NKC^KD^ mice were collected on day 5 p.i., weighed, and virus titer (presented as PFU/g of lung tissue) was assessed in lung homogenates by plaque assay on MDCK cells. **(D)** Schematic representation of NK cell education/licensing and pathogen recognition by NK cells that express self-MHC-I-specific inhibitory Ly49 (licensed) and those that do not (unlicensed).

We wanted to address whether perforin or IFN-γ protects NKC^KD^ mice against influenza virus infection. To determine whether the absence of perforin can be fatal for NKC^KD^ mice, we crossed NKC^KD^ mice to perforin-deficient mice (*Prf* ^-/-^) on the C57BL/6 background to obtain NKC^KD^-Prf^-/-^ mice. We inoculated NKC^KD^ and NKC^KD^-Prf^-/-^ mice with influenza virus intranasally. Interestingly, in contrast to NKC^KD^ mice, all NKC^KD^-Prf^-/-^ mice succumbed to the influenza infection (Fig 7A). Similarly, to address the role of IFN-γ, two groups of NKC^KD^ mice were inoculated with influenza virus intranasally. We neutralized IFN-γ in one group of influenza-infected mice using anti-IFN-γ mAb injections two days prior to and at the day of influenza infection, and every two days until day 10 p.i. As a control, a group of uninfected NKC^KD^ mice were treated with anti-IFN-γ mAb as well. Almost 90% of influenza virus-infected NKC^KD^ mice that were treated with anti-IFN-γ mAb died from the infection (Fig 7B). Both the NKC^KD^-Prf^-/-^ and anti-IFN-γ mAb-treated NKC^KD^ mice had higher lung viral titers compared to the NKC^KD^ control group on day 5 and 7, prior to the engagement of an adaptive immune response (Fig 7C). These data show that protective NK cell-mediated antiviral defenses in NKC^KD^ mice during influenza virus infection require both IFN-γ and perforin.

## Discussion

In the current study, we demonstrate for the first time that disrupting the interaction between MHC-I and Ly49 inhibitory receptors on NK cells protects mice from lethal influenza virus infection. Here, we found that the proportion of lung epithelial cells that express MHC-I increased dramatically following influenza virus infection, in an IFN-I independent manner. We also provide evidence for the relevance of this upregulation in the severity of influenza virus infection, in which upregulation of MHC-I on lung epithelial cells in the presence of Ly49:MHC-I interactions allows influenza virus to escape recognition by NK cells, facilitating viral replication and mouse mortality. This demonstrates the direct contribution of Ly49:MHC-I interactions in the severity of influenza virus infection.

The human analogues of the mouse Ly49 receptors are the KIRs. Previous studies have reported that patients with either mild or severe pandemic influenza A H1N1/2009 virus infections had significantly higher levels of inhibitory KIR2DL5 gene expression in comparison to healthy individuals [40]. The presence or absence of interactions between NK cells expressing the KIR2DL5 inhibitory receptor and targets expressing its MHC-I ligand could be responsible for the severity of influenza virus infection in human patients. Moreover, another group observed that inhibitory KIR2DL2 and KIR2DL3 allotypes and their cognate ligands, HLA-C1 and HLA-C2, respectively, are significantly enriched in H1N1/2009 intensive-care unit patients in comparison to healthy individuals [41]. Taken together, these reports suggest that potential associations of specific inhibitory KIRs and their MHC-I ligands during severe influenza A virus infections may result in decreased NK function and increased virus pathogenicity. *In vitro* studies have shown that impairment of human NK cell activity is directly mediated by the accumulation of MHC-I molecules on the surface of influenza virus-infected cells, which in turn increases binding of the NK cell inhibitory receptor KIR2DL1 to the infected cells and inhibits human NK cell-mediated cytotoxicity against them *in vitro* [11, 12]. MHC-I upregulation by influenza virus may act as a mechanism to evade NK cell recognition by inhibiting NK cell functions via inhibitory Ly49 engagement. Disrupting this interaction between MHC-I and inhibitory NK cell receptors in humans may interfere with this and reduce the severity of an infection.

Ly49-defecient mice are able to survive an influenza virus infection specifically due to the lack of Ly49 inhibitory receptors; transgenic expression of a self-MHC-I-specific inhibitory Ly49 receptor in NKC^KD^ mice restores their influenza susceptibility to WT levels. This demonstrates the direct contribution of Ly49:MHC-I interactions in the severity of influenza virus infection. The presence of Ly49I restored the Ly49:MHC-I interaction in NKC^KD^ NK cells, and lead to significant mortality. While licensed NK cells are protective against MHC-I-deficient tumors [22], we show that unlicensed NK cells are chiefly responsible for protection from influenza, which may also be true with other viral infections. This could be explained by the lack of inhibitory Ly49 receptors which, if engaged by their MHC-I ligands on the virus-infected cells, could otherwise result in NK cell inhibition (Fig 7D). This hypothesis is supported by our F(ab’)_2_ fragment-mediated Ly49C/I blockade, which was found to be protective in WT mice against influenza virus infection, possibly by disrupting the Ly49C/I:MHC-I interaction. Protection conferred by the blocking of Ly49C/I receptors was smaller compared to the lack of Ly49 receptors (NKC^KD^) or MHC-I ligands (*B2m* ^-/-^), possibly because of a suboptimal blocking in these experiment or the involvement of other inhibitory MHC-I receptors in influenza immunoevasion.

Our results from influenza virus infection in NKC^KD^ mice indicate that survival is associated with decreased cytokine and chemokine levels. It would be reasonable, therefore, to assume that unlicensed NK cells are ‘protective’ by being hyporesponsive, leading to a reduced immune response and consequentially less immune-mediated tissue damage. If this were true, a reduction in NK cell activity would be protective in mice challenged with influenza. However, our data show that reducing the activity of NK cells in NKC^KD^ mice, either by deleting perforin or by neutralizing IFN-γ, is detrimental to the animal’s survival. One caveat to interpreting these data is that neither model is NK specific, and T cells will also be affected by a loss of perforin or IFN-γ. As we have shown, however, NK depletion in NKC^KD^ mice is sufficient to disrupt their viral resistance, making it difficult to discount NK-derived perforin and IFN-γ. Furthermore, although infected with equal amounts of virus, perforin-deficient and IFN-γ-neutralized NKC^KD^ mice show a significantly higher viral titer compared to the NKC^KD^ control mice on days 5 and 7 p.i., before a T cell response is established. In addition, the NKC^KD^ mice show a significantly reduced viral titer compared to the WT mice on day 5 p.i., implying that the NK cells in these mice are more efficient in clearing the virus. Taken together, these results indicate that the protection enjoyed by NKC^KD^ mice is not due to a decrease in NK cell activity but is in fact due to an increased efficiency at clearing the virus. This is also supported by increased viral lethality following the depletion of NK cells in *B2m* ^-/-^ mice. This increased efficiency of unlicensed NK cells against influenza agrees with previous results obtained with MCMV infections [28]. Our survival data indicate that the NK cells in the NKC^KD^ mice are more protective than in WT, suggesting that what we have called ‘unlicensed’ NK cells do in fact possess an efficient anti-viral activity. This could explain why, in a normal mouse immune system, up to half of the NK cells are ‘unlicensed’ with regard to self-Ly49 expression [18, 25]. If the two compartments were understood to provide immunity under different circumstances (tumor versus infection), there is a rationale for maintaining such a large pool of NK cells that, until now, were believed to be dysfunctional.

We propose that the increased efficiency of NK cells in clearing influenza virus in NKC^KD^ mice results in less need for an extended and toxic immune response, reducing the amount of inflammatory cytokines known to cause severe lung damage and a high rate of mortality during influenza virus infection [38, 42]. Survival of MHC-I-deficient mice infected with influenza virus was previously attributed to the lack of cytotoxic CD8^+^ T cells and hence reduced pulmonary damage in these mice [43]. Our data, on the other hand, show a dominant role for NK cells in the protection of these mice against influenza virus infection, since depletion of NK cells makes them susceptible. Consistent with our observations is the reported reduced frequency of NK cells in patients with severe H1N1/2009 infections; CD8^+^ effector T cells were detected at normal levels in these patients [44].

Our study identifies the mechanisms by which influenza virus escapes recognition by NK cells. Disrupting this interaction between MHC-I and inhibitory NK cell receptors *in vivo* interferes with this evasion mechanism and, thus, alters the severity of the infection. Additionally, we have demonstrated a previously unidentified role for unlicensed NK cells of the innate immune system. These observations open up a new field of investigation related to NK cell education and encourage more precise elucidation of the nature of each of these functional NK subsets.

## Supporting Information

S1 Fig Validation of anti-asialoGM1 depletion in NKC^KD^ mice. **(A)** Flow cytometry was performed as in Fig 1 on cells from uninfected or infected NKC^KD^ mice to determine degree of NK-specific depletion in anti-AsialoGM1 treated animals. The proportion of TCRβ^+^ cells is indicated on each plot to show no effect of anti-AsialoGM1 treatment on the T cell compartment. A timeline of injections for this validation is also shown. **(B)** Timeline showing injection series and volume of anti-AsialoGM1 treatment of mice in Fig 1F.
(EPS)

S2 Fig Survival and histopathology in WT and NKC^KD^ mice infected with influenza virus. (A) Groups of age- and sex-matched WT and NKC^KD^ mice (n = 5) were infected with influenza virus (50 and 5000 PFU) and monitored for 2 weeks. (B) WT and NKC^KD^ mice were inoculated intranasally with 1050 PFU of influenza virus. Lungs were collected from WT and NKC^KD^ mice 7 days p.i., fixed in neutral-buffered 10% formalin, sectioned, and stained with H&E. Images were acquired at 100x magnification. One representative image from each group is shown.
(EPS)

S3 Fig Expression of NKG2A and NKG2A/C/E on WT, NKC^KD^, and *B2m* ^-/-^ mice. NK cells were collected, processed, and analyzed as in Fig 1. The NKG2A-specific antibody was raised in 129-background mice, and so cannot be used in our NKC^KD^ mice. Data are pooled from two independent experiments, n = 6 mice per group. Mean ± SEM is shown, with *p< 0.05, **p< 0.01 and ***p< 0.001. Statistical analysis was performed by one-way ANOVA followed by a Bonferroni post-hoc test.
(EPS)

S4 Fig Intracelluar IFN-γ levels and CD107a expression on NK cells from poly (I:C)-treated and influenza-infected mice. Mice were either injected with 15 μg poly (I:C) i.p. for 18 h or infected with influenza virus for 3 days. Cells isolated from spleen and lungs were unstimulated, co-cultured with YAC-I cells at 1:1 cell ratio, or cultured with 10 μg/ml PMA + 1 μg/ml ionomycin in presence of brefeldin A for 4 h. Intracellular IFN-γ (**A**) and CD107a (**B**) staining was performed as in the Materials and Methods. Data are pooled from two independent experiments. Statistical analysis was performed by two-tailed Student’s *t*-test. *p < 0.05, **p < 0.01 and ***p < 0.001.
(EPS)

S5 Fig No difference in IFN-γ production by uneducated or educated NK cells from influenza infected lungs. Cells were stained, processed, and analyzed as in Fig 1. IFN-γ expression was determined after four hours of **(A)** culture in RPMI supplemented with 10% FBS, penicillin/streptomycin, HEPES, non-essential amino acids, 2-mercaptoethanol, and brefeldin A, **(B)** co-culture as in **(A)** with YAC-1 target cells at 1:1 cell ratio, or **(C)** culture as in **(A)** with 10 μg/ml PMA + 1 μg/ml ionomycin. Data are pooled from two independent experiments (n = 6 mice in each), and mean ± SEM is shown. Statistical analysis was performed by one-way ANOVA.
(EPS)

S6 Fig Similar expression of IFN-γ and CD107a on WT, NKC^KD^, and *B2m* ^-/-^ mice. Cells from infected WT, NKC^KD^ **(A, B)**, and *B2m* ^-/-^ **(C, D)** mice were collected, processed, co-cultured, and analyzed as in S3 Fig. Results are pooled from two independent experiments, with each symbol representing one mouse. Horizontal bars represent means, with *p< 0.05. Statistical analysis was performed by a two-tailed Student’s t-test.
(EPS)

S7 Fig No difference in early or late viral titer between WT, NKC^KD^, and *B2m* ^-/-^ mice. Virus titer was determined as in Fig 3. **(A)** No difference in early or late viral titers between WT and NKC^KD^ mice. **(B)** No difference in viral titer on day 5 or day 7 p.i. between WT and *B2m* ^-/-^ mice. Each symbol represents one mouse. Horizontal bars represent mean. Statistical analysis was performed by a two-tailed Student’s t-test, with no significant differences found.
(EPS)

S8 Fig Production of cytokines and chemokines in the lungs of influenza virus-infected mice. Groups of age and sex-matched WT and NKC^KD^ mice were infected intranasally with 600 PFU of FM-MA virus. Lungs were collected at day 5 p.i., and lung tissue homogenates were prepared. The levels of IL-1α, IL-2, IL-4, IL-5, IL-6, IL-10, IL-13, IL-21, IL-22, IL-27 RANTES and GM-CSF in the lung tissue homogenates were measured by flow cytometry using FlowCytomix Multiplex kits. Data are pooled from three independent experiments. Each symbol represents a single mouse. Statistical analysis was performed using Student’s t-test.
(EPS)
