# Correction: Influenza Virus Targets Class I MHC-Educated NK Cells for Immunoevasion

**DOI:** 10.1371/journal.ppat.1006021

**Published:** 2016-11-04

**Authors:** Ahmad Bakur Mahmoud, Megan M. Tu, Andrew Wight, Haggag S. Zein, Mir Munir A. Rahim, Seung-Hwan Lee, Harman S. Sekhon, Earl G. Brown, Andrew P. Makrigiannis

Fig 3 is incorrect. In Fig 3, panels A and B contain the same MHC-I (H2Db) histogram plot for the WT (B6) infected sample. The authors have provided a corrected version here.

**Fig 3:**
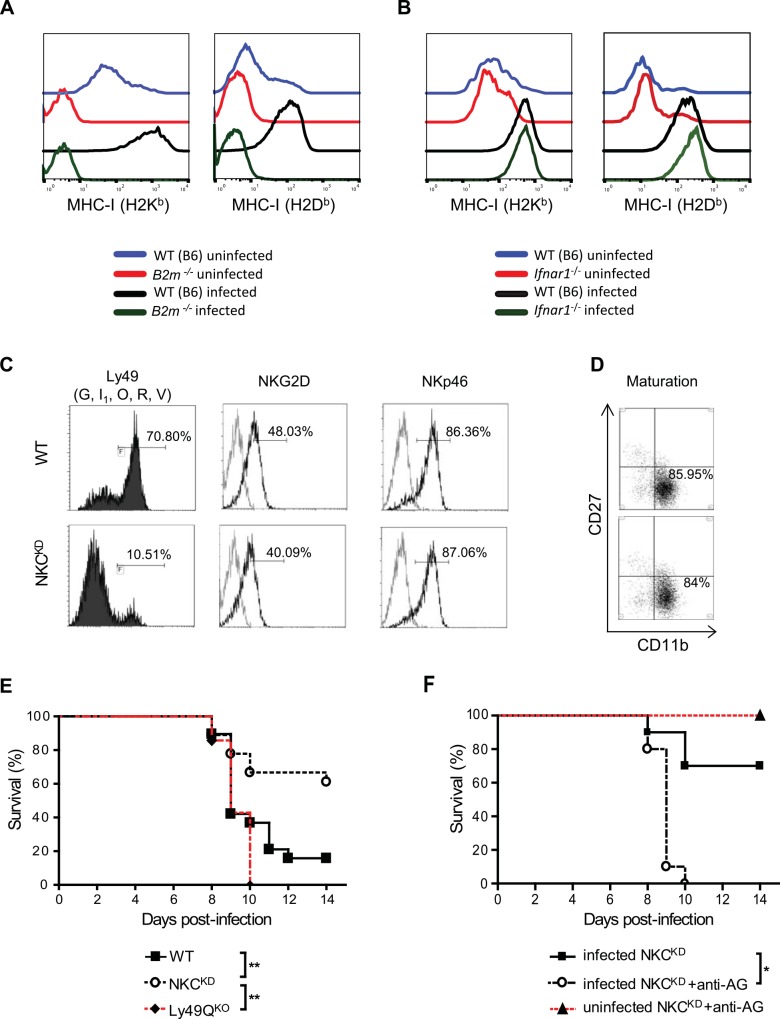
Lung immunopathology of influenza-infected WT and NKCKD mice. (A-D) WT and NKCKD mice were inoculated intranasally with 600 PFU of FM-MA virus. Lungs were collected from WT and NKCKD mice 7 days p.i., fixed in neutral-buffered 10% formalin, sectioned, and stained with H&E. Images were acquired at 100x magnification. One representative image from each group is shown. Images were scored by a pathologist blind to the experimental conditions. Regions of tissue damage are indicated as follows: ‘→’ pulmonary edema; ‘*’ diffuse alveolar damage; ‘‡’ lymphocytic and neutrophilic infiltrate; ‘&’ bronchi filled with cellular debris. This experiment was performed three times with similar results. Two to three mice were used for each group per experiment. (E) Lungs from infected (600 PFU) age- and sex-matched WT and NKCKD mice were collected on day 5 p.i., weighed, and virus titer (presented as PFU/g of lung tissue) was assessed in lung homogenates by plaque assay on MDCK cells. Data are pooled from three independent experiments (n = 10 in each group). Each symbol represents a single mouse. Horizontal bars represent mean values. ***p < 0.001. Statistical analysis was performed using Student’s t-test.
