# Correction: Correction: Correction: Influenza Virus Targets Class I MHC-Educated NK Cells for Immunoevasion

**DOI:** 10.1371/journal.ppat.1006210

**Published:** 2017-02-15

**Authors:** The PLOS Pathogens Staff

There is an error in the correction published on December 16, 2016. The incorrect figure legend was included in the correction. The correct figure legend and Fig 1 are provided here.

The publisher apologizes for the error.

**Fig 1:**
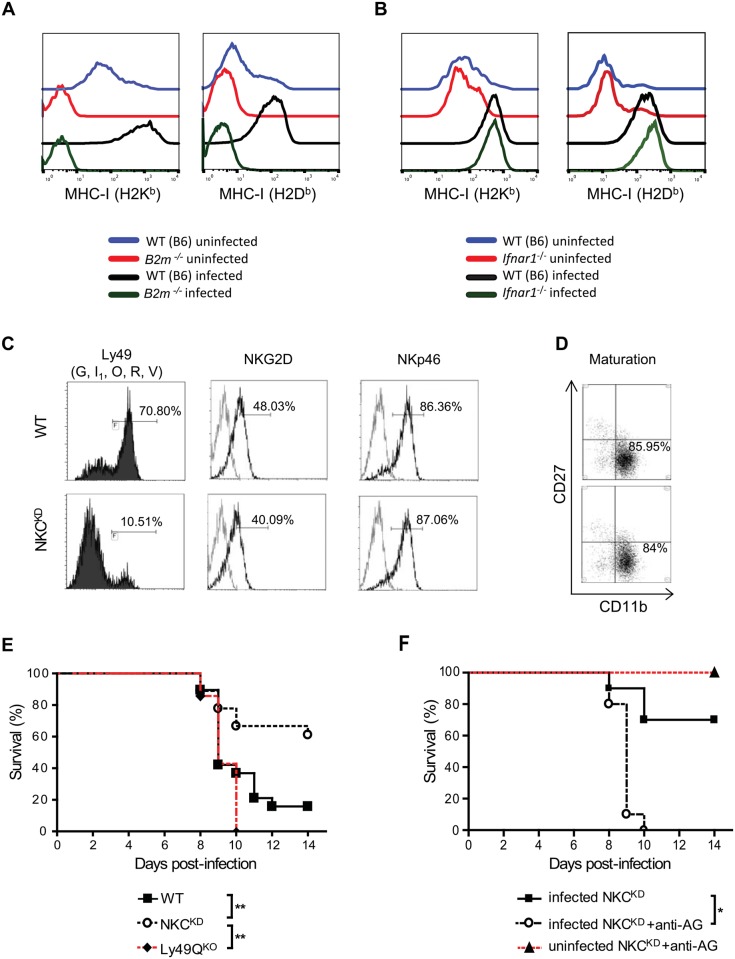
Ly49-deficient mice are protected from lethal influenza infection. **(A, B)** Groups of sex-matched WT (B6), Ifnar1-/- and B2m-/- mice were infected intranasally with 600 PFU of FM-MA virus. Single-cell suspensions were prepared from uninfected lungs and those infected with the virus for 5 days. Cells were stained with antibodies against H-2Kb or H-2Db and CD326 (EpCAM—epithelial cell marker), and analyzed by flow cytometry. Surface expression of H-2Kb or H-2Db was determined on EpCAM+ lung epithelial cells. The following mAb were used in this experiment: anti-LFA-1, anti-EpCAM, anti-H-2Kb, and anti-H-2Db. One representative image from each group is shown. This experiment was performed three times with similar results. **(C, D)** Ly49G, I1, O, R, and V expression was detected on lung NK cells of uninfected WT and NKCKD mice using anti-NKp46, anti-TCRβ, and a combination of 4D11, 4E5 and 14B11 mAb. NKG2D, NKp46, CD11b, and CD27 expression was detected on lung NK cells, defined using anti-CD49b (DX5) and anti-TCRβ. The gray line represents staining with an isotype antibody. This experiment was performed three times with similar results. **(E)** Groups of age and sex-matched WT, Ly49QKO, and NKCKD mice were infected with FM-MA virus (1050 PFU) and monitored for 2 weeks. Data are pooled from two independent experiments (n = 19 in each group). **(F)** Groups of age and sex-matched NKCKD mice with or without NK depletion by anti-asialoGM1 were infected and monitored as above. A group of uninfected, NK-depleted mice was included as a control (n = 10 in each group). The percentage of surviving mice is shown. *p < 0.05, **p < 0.01 and ***p < 0.001. Statistical analysis was performed with the log rank test.
